# Prevalence of Microalbuminuria in Adult Patients with Sickle Cell Disease in Eastern Saudi Arabia

**DOI:** 10.1155/2018/5015764

**Published:** 2018-02-27

**Authors:** Ahmed M. Alkhunaizi, Adil A. Al-Khatti, Mansour A. Alkhunaizi

**Affiliations:** ^1^Nephrology Unit, Specialty Medicine Department, Johns Hopkins Aramco Healthcare, Dhahran, Saudi Arabia; ^2^Hematology Unit, Cancer Institute, Johns Hopkins Aramco Healthcare, Dhahran, Saudi Arabia; ^3^Royal College of Surgeons in Ireland, Dublin, Ireland

## Abstract

**Background:**

Proteinuria is a common feature of sickle cell nephropathy (SCN) that can progress to renal insufficiency and end stage renal disease. Microalbuminuria (MA) is the earliest manifestation of SCN and precedes the development of overt proteinuria. In addition to the renal consequences, MA is linked to cardiovascular complications. Periodic screening and early detection of MA allow early intervention that may reduce the risk of progression to advanced renal failure and cardiovascular diseases.

**Objective:**

The aim of this study was to investigate the prevalence of MA in patients with SCD in the eastern region of Saudi Arabia.

**Methods:**

A prospective cross-sectional observational study was conducted at Johns Hopkins Aramco Healthcare (JHAH). Urine samples of SCD patients 18 years old and older were tested for the presence of MA using urinary albumin over creatinine ratio (ACR). Correlation was tested with multiple variables including age, gender, body mass index (BMI), hemoglobin level, blood pressure, blood transfusion history, pain episodes, and use of hydroxyurea.

**Results:**

Urine samples were tested on 72 patients. The mean age of the study cohort was 35 ± 16.9 years. Microalbuminuria was detected in 18 patients (25%). No correlation was found with any of the tested variables.

**Conclusion:**

Microalbuminuria is a common finding in patients with SCD in eastern Saudi Arabia. Patients with SCD should be screened for MA, and those with positive tests should probably be treated with antiproteinuric agents that may slow the progression to advanced stages of renal failure and decrease the risk of cardiovascular diseases.

## 1. Introduction

Sickle cell disease (SCD) is prevalent in the eastern region of Saudi Arabia (SA). Proteinuria is a common feature of sickle cell nephropathy (SCN) that can progress to renal insufficiency and end stage renal disease (ESRD) [1–3]. Prior to the development of overt proteinuria, patients with SCD manifest with microalbuminuria (MA) that is believed to be a result of hyperfiltration and early glomerular dysfunction [4]. In addition, MA is predictive of all cause and cardiovascular mortality among patients with diabetes mellitus, hypertension, and the general population [5]. The prevalence of MA among patients with SCD in eastern SA has not been previously determined. The objective of this study was to investigate the prevalence of MA and to establish clinical characteristics associated with MA among patients with SCD in this part of the world who have a less severe disease compared to other populations. Early intervention, when MA is present, using antiproteinuric measures such as blockade of the renin angiotensin system or use of hydroxyurea, may slow the progression to overt proteinuria and advanced chronic kidney disease (CKD) [6]. Treatment of MA may also affect mortality and morbidity associated with cardiovascular diseases.

## 2. Methods

A prospective cross-sectional observational study was carried out at Johns Hopkins Aramco Healthcare (JHAH) in Dhahran, eastern SA, between July 2014 and October 2016. Consecutive patients with SCD, 18 years and older, who attended the hematology clinic for routine follow-up at the Cancer Institute of JHAH were enrolled after they gave an informed consent. The diagnosis of SCD was confirmed using hemoglobin electrophoresis. The two genotypes encountered and considered as having SCD were homozygous SS and compound heterozygous sickle-beta zero thalassemia. Patients with diabetes mellitus, essential hypertension, patients who had positive proteinuria by urine dipstick, patients with preexisting renal disease with or without proteinuria, and those who had an acute illness, fever, or SCD crisis in the preceding two weeks were excluded. Pregnant women were also excluded. Patients were instructed to submit early morning urine samples for measurement of MA. In addition to urine samples, blood was collected for measurement of serum creatinine. Estimated glomerular filtration rate (eGFR) was calculated from serum creatinine, age, weight, and gender, using the Cockroft-Gault formula [7]. Data about patients' weight, body mass index (BMI), blood pressure (BP), hospitalization, blood transfusion history, and use of hydroxyurea were collected by reviewing the electronic medical records and reflected the most recent measurements. Baseline fetal hemoglobin (HbF) prior to initiation of hydroxyurea and glucose-6-phosphate dehydrogenase status (G6PD) were also assessed. Cut-offs of the independent variables were determined based on the recognized normal values for BP, BMI, eGFR, and hemoglobin. The mean of three resting systolic and diastolic BP measurements during routine clinic visits was used. Microalbuminuria was determined by measuring the albumin/creatinine ratio (ACR) on an early morning spot urine sample using rate nephelometry. Level of >24.9 mg albumin/g creatinine was considered abnormal. Correlation was made with various variables including age, sex, BP, BMI, level of hemoglobin, use of hydroxyurea, and hospitalization for pain crises. Patients' parameters were divided into age < 30 and ≥30 years; BMI < 25 and ≥ 25 kg/m^2^; SBP < 120 and ≥ 120 mmHg; DBP < 80 and ≥ 80 mmHg; pain crisis < 2 and ≥ 2 episodes/year; Hb < 10 and ≥ 10 g/dL.

The study was approved by the institutional review board at JHAH before it began.

### 2.1. Statistical Analysis

Data were entered into Microsoft Excel 2013 and then uploaded into Statistical Package for Social Sciences (SPSS) 21 for analysis. Association between the independent variables and MA was assessed by bivariate and multivariate analyses using Chi square test (*χ*^2^) for independence and logistic regression analysis.* T*-test was used to compare the mean values. Means were expressed as ±1 standard deviation (SD). Odds ratios (OR) were expressed together with 95% confidence interval (CI) and associated *P* values. A *P* value of < 0.05 was considered significant.

## 3. Results

One hundred and two patients were recruited, of whom 72 submitted urine and blood samples for analysis and were included in the final analysis. The remaining 30 patients (29%) failed to submit urine and or blood samples and were excluded. Out of the 72 patients who were included in the analysis, 37 patients were females (51%) and 35 were males (49%) with a female : male ratio of 1.06 : 1. The vast majority of patients are expected to have the Arab-Indian (AI) beta-globin gene (HBB) cluster haplotype as previously determined [8–10]. The mean age of the study population was 35 ± 16.9 years. The mean BMI of the cohort was 24 ± 4.19 kg/m^2^. The mean SBP and DBP were 118 ± 13 and 72 ± 24 mm Hg, respectively. The mean Hb was 10.5 ± 1.6 g/dL. The mean eGFR was 127 ± 40 ml/min. Seventy (97%) patients were receiving hydroxyurea at the time of enrolment. None of the patients received treatment with angiotensin converting enzyme inhibitors or angiotensin receptor blockers at time of enrolment. The demographics of patients are shown in Table 1.

Increased MA was found in 18 patients (25%). In a multivariate logistic regression model of MA, there was no correlation between the presence of MA and any of the variables including age, sex, BP, BMI, level of Hb, number of hospitalizations for pain crisis, and eGFR as shown in Tables 2 and 3. The mean baseline HbF was 18.4 ± 6.3% of total Hb (range 7–39%) in patients with normal albumin excretion compared to 15.4 ± 9.0% (3.5–33%) in those with MA, *P* = 0.138. In addition, in a post hoc analysis we found no correlation between G6PD status and the development of MA.

## 4. Discussion

Sickle cell nephropathy may develop early in the course of patients with SCD and can have a variety of manifestations such as hyposthenuria, hematuria, proteinuria, abnormal urinary acidification, and renal failure [1, 4, 11]. Microalbuminuria is an early manifestation of SCN which is believed to be a consequence of glomerular hyperfiltration [12]. Glomerular hyperfiltration has been implicated in the pathogenesis of glomerular hypertrophy and glomerular sclerosis which is the predominant glomerular lesion found in renal biopsy of patients with SCN [12, 13].

In SA, SCD is found mainly in the eastern, southwestern, and northwestern regions [8, 9]. Patients in the eastern region have less severe disease, attributed to the presence of the AI beta-globin haplotype that results in a much higher HbF level, as compared with those in the south western and north western regions, who have the Benin haplotype [10, 14, 15]. We have previously determined the prevalence of overt proteinuria among patients with SCD in the eastern region of SA which was less than what had been reported in other populations in the United States and Africa [16–18]. We also have studied the course of patients with SCD who developed ESRD and were started on renal replacement therapy (RRT) at two large centers in eastern SA [19]. Among 942 patients with SCD who were followed up at our center between 2003 and 2016, only 11 patients developed ESRD and required RRT [19]. Therefore, we believe that renal involvement is less common and less severe in this patients' population. However, with the prevalence of MA, the early manifestation of SCN in this patients' population has not been previously determined.

The aim of this study was to determine the prevalence of MA in SCD patients in eastern SA and to define the clinical and hematologic correlates of the disease. Microalbuminuria is an important marker of early renal complication, and is a predictor of cardiovascular disease not only among diabetic and hypertensive patients but also in the general population [5].

Unlike many studies that have looked at the prevalence of MA in children with SCD, our cohort included adults above the age of 18 years. We found that the overall prevalence of MA among adult patients with SCD was 25%. In comparison, we have previously determined the prevalence of overt proteinuria (macroalbuminuria) in the same patients' population to be 8.4% [16]. Microalbuminuria cannot be detected by simple urine dipstick testing, and the presence of abnormal albumin excretion should be performed using more sensitive methods such as nephelometry. Adding the prevalence of macroalbuminuria found in our previous study to the current prevalence of MA, we can conclude that the overall prevalence of abnormal albumin excretion among SCD patients in this geographic area approximates 30%. We did not find a correlation between MA and any of the variables studied including age, gender, BMI, BP, level of Hb, frequency of pain episodes, and eGFR. Similarly there was no correlation between the development of MA and the G6PD status, a condition that predisposes to episodic hemolysis among affected individuals. In addition, there was no difference in the level of HbF between patients with MA and those with normal albumin excretion. The relatively small size of the study population may have contributed to the lack of correlation.

The mean eGFR in our cohort is relatively high and is likely to be an overestimate of the true GFR. We used the Cockcroft-Gault formula to estimate GFR which was derived from estimation of creatinine production based on gender, age, and weight [7]. Cockcroft-Gault formula may overestimate creatinine production in patients with SCD who have low muscle mass. In addition, proximal tubular excretion of creatinine in patients with SCD is believed to be elevated, averaging 40% even in the presence of normal renal function which may lead to over estimation of GFR [20]. Other methods to measure GFR such as inulin and iothalamate clearance are more accurate albeit cumbersome and not practical to use in every day practice.

Other studies have shown a higher prevalence of albuminuria among African American patients with SCD. In an earlier study in African American children, Dhrnidharka et al. found MA in 46% of children between 10 and 18 years of age [21]. There was a correlation with age but no correlation with pain frequency, hospitalization, frequency of blood transfusion, ferritin level, and creatinine clearance. Guasch et al. investigated the prevalence of albuminuria (micro- and macroalbuminuria) among African American adults with SCD [17]. In hemoglobin SS disease, increased albuminuria occurred in 68% of adult patients, and macroalbuminuria occurred in 26%. At the age of 40 years, 40% of patients with SS disease had macroalbuminuria. The prevalence of albuminuria was more common in SS disease compared with other sickling disorders. Albuminuria correlated with age and serum creatinine in SS disease but not with BP or hemoglobin levels. In a more recent study, MA was found in 44% of adults with HbSS as compared to 23% in patients with HbSC [22].

Other investigators found MA in 40% of teenagers and adults with SCD in Brazil with no correlation between MA and age, creatinine clearance, and Hb level [23]. In a cohort study in Jamaica, MA was found in 26% of subjects with SCD between the ages of 18 and 23 years. There was a positive correlation with GFR and BP and a negative correlation with Hb [24].

In our cohort, the great majority of the patients were receiving hydroxyurea at the time of enrolment. Whether treatment with hydroxyurea has affected the course of patients and lowered the prevalence of MA is not known. The data about the role of hydroxyurea in modifying the renal abnormalities associated with SCD are conflicting. McKie et al. have shown that microalbumin excretion normalized in 44% of patients with SCD treated with hydroxyurea [6]. In a more recent study, Aygun et al. reported that, after three years of treatment with hydroxyurea, there was a decrease in hyperfiltration, and the GFR dropped from 167 to 145 ml/min; however, there was no change in urine microalbumin excretion [25]. Similarly, the BABY HUG clinical trial for infants with SCD showed that treatment with hydroxyurea for 24 months did not influence GFR in young children with SCD. However, hydroxyurea was associated with better urine concentrating ability and less renal enlargement [26]. Considering the good safety profile of hydroxyurea and the benefits in patients with SCD in general, we believe that all patients with SCD who have MA should be treated with hydroxyurea in addition to blockers of the renin angiotensin system. Treatment with captopril was shown in a small study of 22 patients to be effective in reducing albuminuria associated with SCD [27]. Similarly, in a phase-2 multicenter trial, losartan decreased urinary albumin excretion in SCD patients with albuminuria, especially in those with MA [28]. More recently, losartan was shown to decrease albuminuria in 20 patients with SCD who were treated with hydroxyurea [29]. A larger prospective randomized study will be required to document the effectiveness of renin angiotensin system inhibitors to decrease MA in this patients' population.

In conclusion, MA is an early manifestation of SCN and is a common finding in patients with SCD in eastern SA. All patients with SCD should be screened periodically for MA and those who test positive may benefit from treatment with antiproteinuric agents in addition to hydroxyurea.

## Figures and Tables

**Table 1:** Patients' demographics.

Total	Male (%)	Female (%)	Age Years	BMI Kg/m^2^	SBP mmHg	DBP mmHg	Pain crisis	eGFR ml/min	Hb g/dL	HbF%	HU (%)
72	35 (49)	37 (51)	35 ± 16.9	24.2 ± 4.19	118 ± 13	76 ± 24	2	127 ± 40	10.5 ± 1.6	17.6 ± 7.1	70 (97)

BMI: body mass index; SBP: systolic blood pressure; DBP: diastolic blood pressure; eGFR: estimated glomerular filtration rate; Hb: hemoglobin; HbF: fetal hemoglobin; HU: hydroxyurea.

**Table 2:** Study variables in association with microalbuminuria.

Variable	Abnormal ACR *N* = 18 (25%)	Normal ACR *N* = 54 (75%)	*χ* ^2^ value	*P* value
	No (%)	No (%)		
Sex				
Male	10 (28.6)	25 (71.4)	0.463	0.496
Female	8 (21.6)	29 (78.4)		
Age (years)				
<30	9 (26.5)	25 (73.5)	0.074	0.785
≥30	9 (23.7)	29 (76.3)		
BMI (kg/m^2^)				
<25	10 (26.3)	28 (73.7)	0.074	0.785
≥25	8 (23.5)	26 (76.5)		
SBP (mm Hg)				
<120	9 (21.4)	33 (78.6)	0.686	0.408
≥120	9 (30)	21 (70)		
DBP (mm Hg)				
<80	14 (22.6)	48 (77.4)	1.394	0.238
≥80	4 (40)	6 (60)		
Pain crisis				
<2	9 (20.9)	34 (79.1)	0.943	0.331
≥2	9 (31)	20 (69)		
Hb (gm/dL)				
<10	7 (31.8)	15 (68.2)	0.785	0.375
≥10	11 (22)	39 (78)		
eGFR (ml/min)				
<100	6 (33.3)	12 (66.7)	0.889	0.346
≥100	12 (22.2)	42 (77.8)		

ACR: albumin/creatinine ratio; BMI: body mass index; SBP: systolic blood pressure; DBP: diastolic blood pressure; Hb: hemoglobin; eGFR: estimated glomerular filtration rate.

**Table 3:** Logistic regression analysis of study variables in association with microalbuminuria.

Variable	OR	95% CI	*P* value
Sex			
Male versus female	0.402	0.10–1.64	0.204
Age (years)			
<30 versus ≥30	1.618	0.41–6.41	0.494
BMI (kg/m^2^)			
<25 versus ≥25	1.139	0.33–4.00	0.838
SBP (mm Hg)			
<120 versus ≥120	0.858	0.22–3.40	0.827
DBP (mm Hg)			
<80 versus ≥80	0.471	0.09–2.56	0.383
Pain crisis			
<2 versus ≥2	0.467	0.14–1.58	0.219
Hb (gm/dL)			
<10 versus ≥10	2.072	0.52–8.30	0.304
eGFR (ml/min)			
<100 versus ≥100	0.300	0.06–1.63	0.163

OR: odds ratio; CI: confidence interval; BMI: body mass index; SBP: systolic blood pressure; DBP: diastolic blood pressure; Hb: hemoglobin; eGFR: estimated glomerular filtration rate.
